# The impact factor of an open access journal does not contribute to an article’s citations

**DOI:** 10.12688/f1000research.10892.1

**Published:** 2017-03-02

**Authors:** SK Chua, Ahmad M Qureshi, Vijay Krishnan, Dinker R Pai, Laila B Kamal, Sharmilla Gunasegaran, MZ Afzal, Lahiru Ambawatta, JY Gan, PY Kew, Than Winn, Suneet Sood

**Affiliations:** 1 Jeffrey Cheah School of Medicine and Health Sciences, Monash University Malaysia, Selangor, Malaysia; 2 Department of Public Health, Jeffrey Cheah School of Medicine and Health Sciences, Monash University Malaysia, Selangor, Malaysia; 3 All India Institute of Medical Sciences, New Delhi, India; 4 Ng Teng Fong General Hospital, JurongHealth, Jurong East, Singapore; 5 Department of Community Medicine, MAHSA University, Kuala Lumpur, Malaysia; 6 Department of Surgery, Jeffrey Cheah School of Medicine and Health Sciences, Monash University Malaysia, Selangor, Malaysia

**Keywords:** bibliometrics, bibliometric analysis, information science, publications, literature based discovery, open access, Web of Science, Google Scholar

## Abstract

Background

Citations of papers are positively influenced by the journal’s impact factor (IF). For non-open access (non-OA) journals, this influence may be due to the fact that high-IF journals are more often purchased by libraries, and are therefore more often available to researchers, than low-IF journals. This positive influence has not, however, been shown specifically for papers published in open access (OA) journals, which are universally accessible, and do not need library purchase. It is therefore important to ascertain if the IF influences citations in OA journals too.

Methods

203 randomized controlled trials (102 OA and 101 non-OA) published in January 2011 were included in the study. Five-year citations for papers published in OA journals were compared to those for non-OA journals. Source papers were derived from PubMed. Citations were retrieved from Web of Science, Scopus, and Google Scholar databases. The Thompson-Reuter’s IF was used.

Results

OA journals were found to have significantly more citations overall compared to non-OA journals (median 15.5 vs 12, p=0.039). The IF did not correlate with citations for OA journals (Spearman’s rho =0.187, p=0.60). The increase in the citations with increasing IF was minimal for OA journals (beta coefficient = 3.346, 95% CI -0.464, 7.156, p=0.084). In contrast, the IF did show moderate correlation with citations for articles published in non-OA journals (Spearman’s rho=0.514, p<0.001). The increase in the number of citations was also significant (beta coefficient = 4.347, 95% CI 2.42, 6.274, p<0.001).

Conclusion

It is better to publish in an OA journal for more citations. It may not be worth paying high publishing fees for higher IF journals, because there is minimal gain in terms of increased number of citations. On the other hand, if one wishes to publish in a non-OA journal, it is better to choose one with a high IF.

## Introduction

A journal’s impact factor (IF) has long been used as a measure of the quality of a journal ^1^. Today, the IF is used as a tool to assess researchers for employment, career promotion, and funding ^2– 4^.

In the past, most libraries could possess only a limited number of journals, and librarians used the IF to decide which journals to buy ^3, 5– 7^. Consequently, high IF journals were more likely to be purchased, read, and cited. With low IF journals, availability was a constraint. Scientists, wanting a greater audience for their research, preferred to publish in high IF journals. There was plenty of evidence that publishing in a higher IF journal resulted in more citations ^8– 13^.

In contrast, at present, open access (OA) journals are universally available. Libraries have no need to subscribe, and researchers can access OA articles freely. Expectedly, OA publication is associated with increased citations ^14– 19^, so researchers are likely to prefer this path. What is not known is whether, within OA journals, increasing IF is associated with increasing citations, as it is for non-OA journals. Yet this information is important, since cost of publishing in an open access journal is high and increases with the journal’s IF. Should a researcher, or a sponsor, pay good money for publication in a higher IF OA journal if the IF will not influence citations?

We conducted a study to determine whether an OA journal’s IF influences citations.

## Methods

We first conducted a pilot study to estimate required sample size. For this purpose, 57 randomized controlled trials (RCTs) were extracted from PubMed, and scanned for citations as listed in the Web of Science. PubMed was chosen to look for source articles because most researchers start their search on PubMed ^20^. Within this pilot group, for OA articles the mean citations were 12.0±8.81; for non-OA articles the mean citations were 7.14±6.89. The estimated sample size, at α= 0.05 and β=0.2, was 58 articles per group, which we rounded up to an intended 100 articles per group.

In order to have a 5-year follow up for citations, we chose 2011 as the publication year of articles included in this study, and restricted our source articles to those published in January 2011. We found 3,742 RCTs, and saved them into a Microsoft Excel file. The IF of their journals were derived from the Thompson Reuters’ Web of Science database.

From these 3,742 articles, we extracted titles until at least 100 articles met the criteria for OA, and 100 for non-OA. Articles were picked at random, using MS Excel’s RANDBETWEEN function.

Articles were considered OA if the journal title was present in PubMed’s OA subset list as open access, and open access was allowed immediately upon publication.

Articles were considered non-OA if the following three conditions were all fulfilled:

1. The publishing journal was not listed in PubMed’s OA subset list;

2. The article was never made freely available by the journal;

3. The article was not self-archived (as determined by a careful web search for the article).

**Figure 1.**
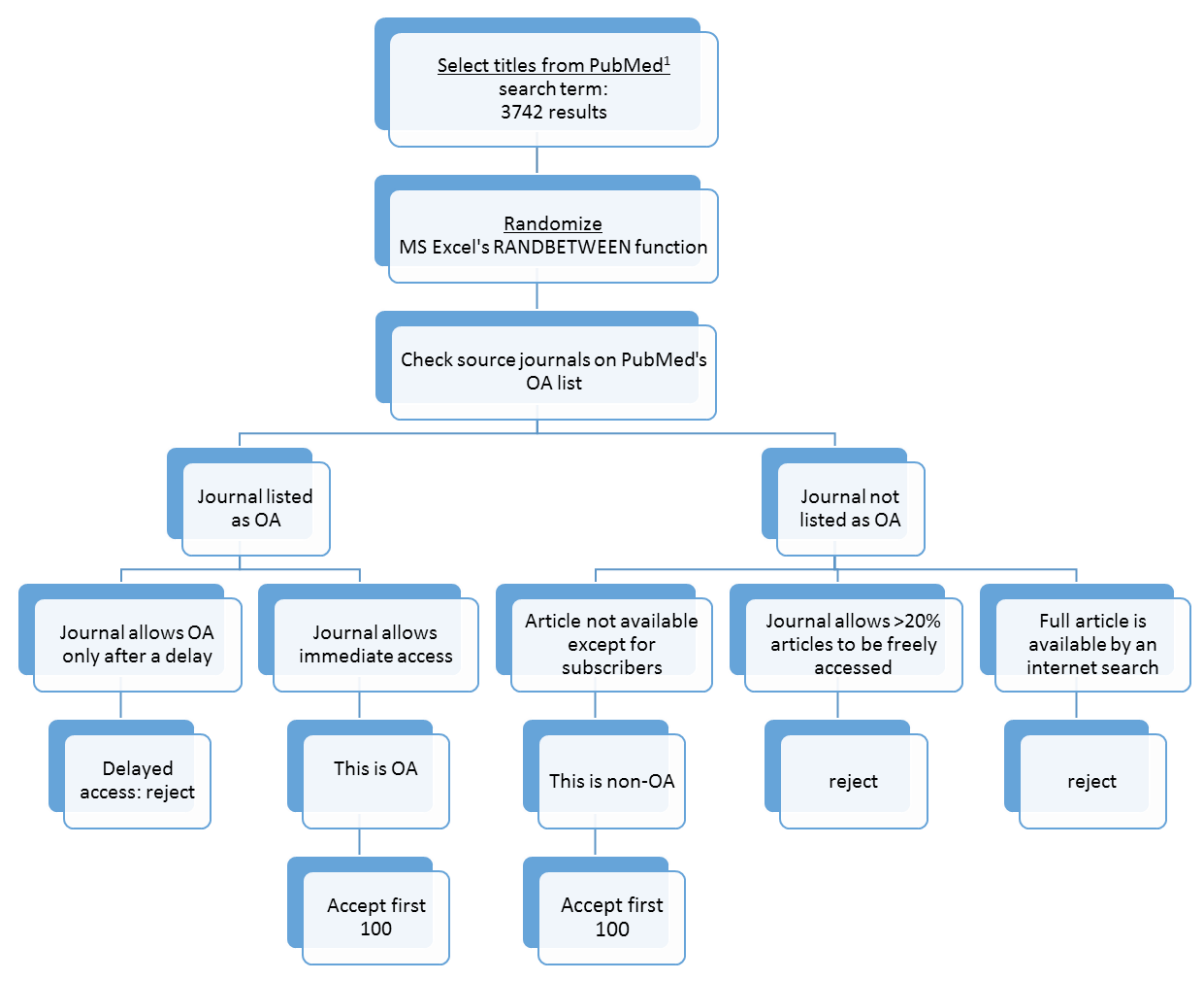
Workflow of OA and non-OA article selection from PubMed. ^1^We entered the PubMed search command: “Randomized Controlled Trial [ptyp] AND (“2011/01/01“[PDAT] : “2011/01/31“[PDAT])”. Ptype = publication type, pdat = publication date.

In other words, the non-OA article could, in theory, only be read by someone with a subscription. Within non-OA journals, we excluded articles if their journals allowed free access to all articles any time after publication. We further excluded articles published in hybrid non-OA journals if over 20% of their articles were freely available (for this, we counted 100 successive 2011 articles in that journal, and ensured that fewer than 20 were marked as freely accessible). In other words, we attempted to ensure that the non-OA journal was true non-OA, and its IF would properly represent the IF of a non-OA journal ( Figure 1). Finally, we also excluded articles if their journal did not have a measurable Web of Science IF for 2011.

The articles were scanned for citations as listed in Web of Science, Scopus, and Google Scholar databases. The search period was extended up to 2016, allowing for five years of publication time, with the assumption that citations over five years provide a better estimate of the impact of a paper than citations over two years ^5^. Only journal citations were included in the counts; citations in books, theses, and government documents were excluded to conform with the Web of Science policy ^21^. We exported citation data from the three databases into .csv files, and imported these into a Microsoft Excel sheet. Duplicates were excluded. Citations that appeared in two language versions of the same paper were counted as one.

IBM ^®^ SPSS ^®^ Statistics (version 22.0) software was used to conduct the statistical data analyses on the dataset (Dataset 1, doi: 10.7910/DVN/XR6MR9 ^22^). OA journals were compared to non-OA journals for overall IF and citations over 5 years. Normality for each independent variable and dependent variable was assessed using the “Kologorov-Smirnov” test, which showed that citations were not normally distributed (p< 0.05). Consequently, non-parametric univariate analysis was carried out using the “Mann-Whitney” test. Linear regression was performed before and after logarithmic transformation of the data.

## Results

### Citations for articles published in OA and non-OA journals

203 articles (101 non-OA, and 102 OA) fulfilled the criteria for inclusion. The IFs of their journals for 2011 (IF-2011) ranged from 0.121 to 10.111 (median 2.083, mean 2.285±1.323). The median of number of citations was 15 (range 0–92).

There were significantly more citations in OA publications than in non-OA publications. The IFs were almost identical ( Table 1).

**Table 1.** Comparison of citations in OA and non-OA publications, for number of citations and IF-2011. IQR: Interquartile range; SD: standard deviation.

		OA	Non-OA	p
Number of citations	Median (IQR)	15.5 (18)	12 (20)	0.039 ^a^
IF-2011	Mean (SD) Median (IQR)	22.37 (+19.3) 2.083 (0.779)	18.13 (+16.9) 1.996 (1.953)	0.097 ^b^ 0.133 ^a^

^a^Mann-Whitney test ^b^Independent samples t-test

### Correlation between IF-2011 and citations

We assessed the correlation between IF-2011 and citations. Since the data was skewed, we used Spearman’s rho (r _s_). The r _s_ value for all papers was 0.387 (p<0.001).

The correlation was assessed separately for OA and non-OA publications. For OA publications the correlation was very small (r _s_=0.187, p=0.060). In contrast, it was significant (r _s_=0.514, p<0.001) for non-OA publications.

### Regression

We calculated the linear regression coefficient between IF and citations. The crude β regression coefficient was 0.297 ( Table 2). We then calculated the regression values separately for OA and non-OA publications. There was very little correlation between IF and citations for OA publications. The five-year citations increased by 3.3 for every unit increase in IF. There was, however, significant correlation between citations and IF in non-OA publications, which showed a rise in five-year citations by 4.3 for every unit increase in IF ( Table 2).

**Table 2.** Association between IF-2011 and citations for articles published in OA and non-OA journals. Final model equation for all citations: 4.093 × (IF-2011) +10.904. Final model equation for OA citations: 3.346 × (IF-2011) + 14.648. Final model equation for non-OA citations: 4.347 × (IF-2011) + 8.291. β: crude regression coefficient. SE: Standard error.

	β (95% CI)	p	Constant (SE)
All	4.093 (2.264, 5.923)	<0.001	10.904 (0.928)
OA	3.346 (-0.464, 7.156)	0.084	14.648 (1.920)
Non-OA	4.347 (2.420, 6.274)	<0.001	8.291 (0.971)

In view of the skew, we repeated the regression analysis after log _10_ transformation of the citation data. The data became normally distributed after transformation. The outcome was roughly similar to the pre-transformation results ( Table 3).

**Table 3.** Association between IF-2011 and citations for articles published in OA and non-OA journals, after log _10_ transformation of the citations. Final model equation for all log _10_ citations: 0.097 × (IF-2011) + 0.926. Final model equation for log _10_ OA citations: 0.066 × (IF-2011) + 1.055. Final model equation for log _10_ non-OA citations: 0.109 × (IF-2011) + 0.839. β: crude regression coefficient. SE: Standard error.

	β (95% CI)	p	Constant (SE)
All	0.097 (0.057, 0.138)	<0.001	0.926 (0.021)
OA	0.066 (-0.008, 0.141)	0.081	1.055 (0.038)
Non-OA	0.109 (0.061, 0.158)	<0.001	0.839 (0.025)

## Discussion

The IF served an important function in the pre-internet era. Libraries needed to decide which journals to buy. With limited budgets, especially in poorer countries, they purchased only a few of the highest IF journals ^7, 23– 25^. In a self-propagating mechanism, the higher IF journals continued to be better read and cited, and were purchased more often. To quote Peter Suber ^24^, “Prestige even feeds prestige. Journal prestige attracts readers, and helps justify library decisions to spend part of their limited budget on a subscription. The growth in readers and subscribers directly boosts prestige.”

With time, the IF became widely used as a measure of the quality of a journal, author, and paper ^21, 24^. Universities rewarded faculty who published in high-IF journals. Promotion and tenure committees, as well as funding agencies, preferred authors who had published papers in high-IF journals ^24^. Researchers thus were driven to publish their best papers in high-IF journals. Instead of the content identifying the journal, the journal began to identify the content.

Today, the game has changed and the efficiency of the internet has lead to the proliferation of OA journals. Libraries do not need to make any choices at all; the reader just needs to decide which paper is relevant and read it. This has diminished at least one purpose served by the IF: to help institutions decide which journals to buy. It also raises two questions. The first is: Are publications in OA journals more likely to be cited than those in non-OA journals? The second is: Will a higher IF lead to more citations?

### Citations in OA and non-OA journals

OA journals are always available to all—this is their advantage over non-OA journals. Consequently, one would expect that an article published in an OA journal would be more easily accessible, more widely read, and therefore more often cited. Research has proved that this is indeed true ^14, 18, 26^.

Our data has also shown that articles published in OA journals are associated with more citations than those published in non-OA journals—by a factor of 1.3. Although statistically significant, this increase in citations was slightly lower than that shown by others. Antelman ^14^, found that open access publications in various specialties (philosophy, political science, engineering, mathematics) were associated with increased citation rates by a factor of 1.45–1.9. Freely accessible articles had 1.5 times higher citation rates than non-OA articles. Kousha and Abdoli ^18^ showed that citation rates of OA publications were higher by a factor of 1.9, giving them a clear advantage. However, these other authors compared OA articles and non-OA articles, rather than OA journals and non-OA journals. Our data is different as it compares the number of citations of publications in OA journals with citations of publications in non-OA journals.

This leads us on to the next question: Is the expectation of more citations with a higher IF being fulfilled?

### Correlation between citations and IF

At the start of the study we had expected to see a significant correlation between IF and the number of future citations, believing that increasing IF indicated improved quality of journal and article. For OA journals the correlation, however, was poor and insignificant (r _s_=0.187, p=0.060). We believe that it is safe to say an OA journal’s IF contributes little to an article’s future citations.

In contrast, the relationship between citations and IF was strong for non-OA publications. Our correlation coefficient for non-OA publications (0.514), closely matched the values reported by Judge *et al* (0.44) ^12^, Piwowar and Vision (0.45) ^27^, Vanclay (0.56) ^11^, and Leimu and Koricheva (0.62) ^28^. Thus, despite using different databases, particularly Google Scholar, the citation rate in our study showed a moderate (yet statistically significant) correlation with the IF in our study. This validates our methods, and strengthens the findings about OA publications.

### Improvement in citations with increasing IF

Linear regression analysis indicated a very real relationship between citations and IF for non-OA publications. The expected citations rise at an approximate rate of one citation per year per rise in impact factor—a change that is consistent with the very definition of the impact factor. This result was quite similar to the findings reported by Vanclay ^11^ and by Perneger ^29^. In contrast, publishing in an OA journal with a higher IF did not result in significantly increased citations. For every 1 unit rise in IF, the data showed a rise of just over 3 citations in five years; using the log _10_ transformed data the rise was even lower at low IFs. We could not compare our results to those of other authors, as we were unable to find a publication that correlated IF with citations exclusively for OA journals.

We are unable to comment on whether any other variable is a better predictor for an article’s citations compared to the IF, since we did not analyze other factors. Nevertheless, it is reasonable to presume that the article’s quality and relevance will influence the citations much more than IF will. Even for non-OA publications, the citations of an article are likely to be strongly influenced by other factors including the quality of the article, and not by the IF alone. This, of course, is well established ^4, 11, 30^.

### Publishing in OA and non-OA journals

Since OA publications are cited more often, it seems logical that a researcher should publish in an OA journal. Should an author search for a high-IF OA journal? An author may reasonably expect about 14 citations in five years, regardless of the IF, and these would rise to about 20 if the OA journal’s IF was 2 (from 11 to 15 if we use the log _10_ transformed data). With a rise in IF from 0 to 4, the total citations would not even double. And unlike non-OA journals, OA journals charge the author, and, in general, the higher the journal’s IF, the higher the cost. BioMed Central journals with IFs higher than 2 typically charge article-processing fees of about 2000 euros. Even if the journal’s IF contributes to a higher readership and citation rate — which is questionable, considering the low r ^2^ value — it is doubtful whether the few extra citations are worth the cost.

In contrast to OA journals, the number of citations for an article published in a non-OA journal with IF of 4 will be thrice as many as those published in a non-OA journal with an IF of 0. So it makes sense to select as high an IF as possible when publishing an article in a non-OA journal, particularly since non-OA journals charge their readers, and not their contributors.

### Strengths and limitations

We have tried to minimize confounders by selecting RCTs published across one specific month, so that all studies have had the same period of citation. Our other strength was to analyze citations in more than one database: Web of Science, Scopus, and Google Scholar. The inclusion of Google Scholar allowed us to include results from a much larger database ^31^, and thus to provide a better representation of citations than would have been possible if we had depended solely on Web of Science or Scopus. We also ensured that non-OA articles were truly non-OA by excluding those that were self-archived and those that were made freely available by the journals. The journals themselves could also be considered truly non-OA, and consequently their IFs could be considered representative of non-OA journals, because we excluded journals that allowed significant numbers of articles to be freely available. We took care to adhere closely to the Web of Science definition of “Impact Factor” ^21^, by manually examining every Google Scholar citation and excluding citations in books, theses, and government documents. We also included citations over the following 5 years, which we believe provides a better estimate of a paper’s IF ^5^.

The main weakness of our study lies in our inability to evaluate the quality of the papers. In ideal circumstances we would have ensured that all papers were of equivalent quality. However this was not feasible. The other potential issue is that inclusion of citations from Google Scholar might allow entry of poor quality publications and predatory publications ^31^. Despite this possibility, we believe that Google Scholar represents an important database, and must not be excluded.

## Conclusions

OA journals attract more citations than non-OA journals. If all other considerations are equal, a researcher should prefer an OA journal to a non-OA journal for publication. If a researcher publishes in an OA journal, the IF does not matter. It is reasonable to select a journal that will publish quickly and cheaply. If a non-OA journals is selected, the researcher should aim to publish in a journal with a high IF.

## Data availability

The data referenced by this article are under copyright with the following copyright statement: Copyright: © 2017 Chua S et al.

Data associated with the article are available under the terms of the Creative Commons Zero "No rights reserved" data waiver (CC0 1.0 Public domain dedication). 

Dataset 1: Impact factor data. doi, 10.7910/DVN/XR6MR9 ^22^
