# Supplementary material for: The impact factor of an open access journal does not contribute to an article’s citations
Source: F1000Res. 2017 Mar 2;6:208. [Version 1] doi: 10.12688/f1000research.10892.1 (PMC5464220; doi:10.12688/f1000research.10892.1)
Supplement: Peer review history

# Peer review: Referee response for version 1

**DOI:** 10.5256/f1000research.11743.r23330

**Published:** 2017-06-07

**Authors:** Samiran Nundy

**Affiliations:** 1 Department of Surgical Gastroenterology and Liver Transplantation, Sir Ganga Ram Hospital, New Delhi, Delhi, India

This is an interesting approach to the vexed question of whether or not it is worthwhile publishing in the now ubiquitous, and commonly regarded inferior, open access journals.

 The authors have convincingly demonstrated that to get more citations it is. Whether or not this will translate into more prestige points towards selection and promotion for e.g.  faculty positions has to be evaluated later. However these are early days to judge this rather novel open access experiment.

I have read this submission. I believe that I have an appropriate level of expertise to confirm that it is of an acceptable scientific standard.

# Peer review: Referee response for version 1

**DOI:** 10.5256/f1000research.11743.r20913

**Published:** 2017-03-15

**Authors:** Eleftherios P Diamandis

**Affiliations:** 1 Department of Pathology and Laboratory Medicine, Mount Sinai Hospital, Toronto, Ontario, Canada; Department of Laboratory Medicine and Pathobiology, University of Toronto, Toronto, Ontario, Canada

This is a very interesting article which compares the effect of impact factor on collected citations of papers published in open access and non-open access journals. The major findings of this paper is that the open access journals attract more citations than non-open access journals and that the impact factor of an open access journal does not significantly affect the number of citations received. On the other hand, it was found that the number of citations increases with impact factor in non-open access journals. These data have implications for authors who wish to publish their research in either open access or non-open access journals, after considering their associated costs.

 I have no suggestions for further changes of this manuscript except that recently, the findings of the authors have been speculated and/or corroborated with additional publications which should be cited.

  

1. In a paper published in Clin Chem Lab Med 2009 ^1^, it has been suggested that the impact factor will go away soon, effectively in full agreement with the finding that at least for open access journals, the impact factor does not affect citations.
2. In a subsequent paper in Clin Chem Lab Med 2013 ^2^, it has been speculated that journals will act as repositories of information and the impact factor will be irrelevant, further corroborating the findings of this paper that the impact factor of open access journals does not affect citations.
3. Also, in a recent paper in BMC 2017 ^3^, it is mentioned that the journal impact factor is under attack and another factor is proposed to assess journal quality. I believe that these 3 papers are very relevant to this contribution and should be briefly mentioned in the discussion and cited appropriately. Otherwise this is a nice contribution to the discussion related to journal impact factor and related themes.

I have read this submission. I believe that I have an appropriate level of expertise to confirm that it is of an acceptable scientific standard.

## References

[1] DiamandisEP: Journal Impact Factor: it will go away soon. *Clin Chem Lab Med*.2009;47(11) : 10.1515/CCLM.2009.327 1317-8 10.1515/CCLM.2009.327 19778288

[2] DiamandisEP: More discussion on journal impact factor. *Clin Chem Lab Med*.2013;51(12) : 10.1515/cclm-2013-0515 2271 10.1515/cclm-2013-0515 23907426

[3] DiamandisE: The Journal Impact Factor is under attack – use the CAPCI factor instead. *BMC Medicine*.2017;15(1) : 10.1186/s12916-016-0773-5 10.1186/s12916-016-0773-5 28088919PMC5238518
